# CrdR function in a curdlan-producing Agrobacterium sp. ATCC31749 strain

**DOI:** 10.1186/s12866-015-0356-1

**Published:** 2015-02-10

**Authors:** Xiaoqin Yu, Chao Zhang, Liping Yang, Lamei Zhao, Chun Lin, Zhengjie Liu, Zichao Mao

**Affiliations:** College of Agriculture and Biotechnology, Yunnan Agricultural University, Kunming, China; National and Local Joint Engineering Research Center for Screening and Application of Microbial Strains, Kunming, China

**Keywords:** *crdR*, Curdlan, Agrobacterium, Transcriptional regulator

## Abstract

**Background:**

*Agrobacterium* sp. ATCC31749 is an efficient curdlan producer at low pH and under nitrogen starvation. The helix-turn-helix transcriptional regulatory protein (crdR) essential for curdlan production has been analyzed, but whether *crdR* directly acts to cause expression of the curdlan biosynthesis operon (*crdASC*) is uncertain. To elucidate the molecular function of *crdR* in curdlan biosynthesis, we constructed a *crdR* knockout mutant along with pBQcrdR and pBQNcrdR vectors with *crdR* expression driven by a *T5* promoter and *crdR* native promoter, respectively. Also, we constructed a pAG with the green fluorescent protein (GFP) gene driven by a curdlan biosynthetic operon promoter (*crdP*) to measure the effects of *crdR* expression on curdlan biosynthesis.

**Results:**

Compared with wild-type (WT) strain biomass production, the biomass of the *crdR* knockout mutant was not significantly different in either exponential or stationary phases of growth. Mutant cells were non-capsulated and planktonic and produced significantly less curdlan. WT cells were curdlan-capsulated and aggregated in the stationery phase. pBQcrdR transformed to the WT strain had a 38% greater curdlan yield and pBQcrdR and pBQNcrdR transformed to the crdR mutant strain recovered 18% and 105% curdlan titers of the WT ATCC31749 strain, respectively. Consistent with its function of promoting curdlan biosynthesis, curdlan biosynthetic operon promoter (*crdP*) controlled GFP expression caused the transgenic strain to have higher GFP relative fluorescence in the WT strain, and no color change was observed with low GFP relative fluorescence in the *crdR* mutant strain as evidenced by fluorescent microscopy and spectrometric assay. q-RT-PCR revealed that *crdR* expression in the stationary phase was greater than in the exponential phase, and *crdR* overexpression in the WT strain increased *crdA, crdS*, and *crdC* expression. We also confirmed that purified crdR protein can specifically bind to the *crd* operon promoter region, and we inferred that *crdR* directly acts to cause expression of the curdlan biosynthesis operon (*crdASC*).

**Conclusions:**

*CrdR* is a positive transcriptional regulator of the *crd* operon for promoting curdlan biosynthesis in ATCC31749. The potential binding region of crdR is located within the −98 bp fragment upstream from the *crdA* start codon

**Electronic supplementary material:**

The online version of this article (doi:10.1186/s12866-015-0356-1) contains supplementary material, which is available to authorized users.

## Background

Microbes can produce diverse extracellular polysaccharides (EPS) for survival in harsh conditions [1]. Curdlan, a water insoluble β-D-1, 3-glucan, can be efficiently produced by *Agrobacterium* sp. ATCC31749 during stressors of low pH and nitrogen starvation [2-4]. Because of its special gel and immunomodulatory properties, curdlan and its derivatives can be used as food additives and in pharmaceutic products [5-7]. β-D-1,3-glucans can be synthesized by bacteria, fungi [8] and plants [9]; however, large-scale curdlan production occurs mainly via fermentation in *Agrobacterium* [3,10], *Rhizobium* strains [11] and *Cellulomonas flavigena* [12]. An efficient curdlan-producing strain, ATCC31749, whose draft genome sequence is more than 95% homologous to the *Agrobacterium tumefaciens* strain C58 (ATCC33970) genome, is regarded as a model organism for elucidating curdlan biosynthetic pathways and regulatory mechanisms [13,14]. Using chemical mutant selection, the curdlan biosynthesis operon (*crd*) was found to contain *crdA*, *crdS*, and *crdC* genes in the ATCC31749 strain [15-17]. Many cultivating conditions including low pH [18], limited nitrogen [19], high dissolved oxygen [20] and adding uracil or cytosine and phosphate salts [21-23] influence curdlan biosynthesis and accumulation. However, how curdlan biosynthesis gene expression is regulated is unclear.

ATCC31750, a mutant strain derived from ATCC31749, had significantly altered intracellular proteins with changes in pH. Specifically, at pH 5.5 (compared to 7.0), key enzymes of curdlan biosynthesis, such as the catalytic subunit of β-1,3-glucan synthase (*crdS*), UTP-glucose-1-phosphate uridylytransferase (*galU*), and phosphoglucomutase (*pgm*) were increased 10, 3, and 17 times, respectively [18]. Intracellular pH changes may activate synthesis of a cellular stringent response signal (p)ppGpp to alter formation of acidocalcisome, which helps maintain intracellular pH and ion homeostasis [24]. To learn how low pH affects curdlan biosynthesis in an ATCC31749 strain, we analyzed genomic sequences of ATCC31749 (access No: AECL01000001–AECL01000095) and that of *Sinorhizobium meliloti* (access No: NC_003047), which is an acid-tolerant, symbiotic nitrogen-fixing strain [25] using BLAST alignment. We found a transcriptional regulator, *PhrR* (access No: NC_003047.1 (445435–445854), expression of this gene increased 5–6 times under conditions of low pH (pH 6.2) in *S. meliloti* [26]. The *PhrR* gene has a homologous counterpart, *AGRO_0435*, in ATCC31749. Both PhrR and AGRO_0435 are helix-turn-helix transcriptional factors of the XRE-family, which includes HipB of *Escherichia coli* (*E coli*), CH00371 of *Rhizobium Leguminosarum* (*R. Leguminosarum*), and PraR of *Azorhizobium Caulinodans* (*A. caulinodans*) (Additional file 1) [27-35]. The existence of an essential curdlan production regulatory locus other than the crd operon—locus II—was suggested by Stasinopoulos’s group [15] DNA sequencing confirmed that the locus II gene encodes a helix-turn-helix transcriptional regulatory protein, crdR, and that *AGRO_0435* is the *crdR* gene [26], Unfortunately, whether crdR acts directly to regulate *crdASC* expression is unclear, so we investigated the role of crdR on *crdASC* transcriptional activation.

## Methods

### Bacterial strains and vectors used

Strains and vectors used are listed in Table 1. *E. coli* strains TG1 and BL21 used for cloning and expression were grown in Luria-Bertani broth (LB). The *Agrobacterium* sp. ATCC31749 strain was cultivated in LB for growth and for curdlan production, in curdlan-producing medium ([w/v], 5% sucrose, 0.005% yeast extract, 0.5% citric acid, 0.27%K_2_HPO_4_, 0.17% KH_2_PO_4,_ 0.01% MgSO_4_, 0.37% Na_2_SO_4_ 2H_2_O, 0.025% MgCl_2_ · 6H_2_O, 0.0024% FeCl_3_ · 6H_2_O, 0.0015% CaCl_2_ · 2H_2_O, and 0.001% MnCl_2_ · 4H_2_O). Culture pH for strain growth was maintained at 7.0 and lowered to 5.5 immediately for curdlan production in a curdlan-producing medium [36]. Primers for PCR amplification designed by DNAMAN software and synthesized by Sangong Biotech (Shanghai, China) are listed in Table 2.

**Table 1:** **Bacterial strains and plasmids used in this study**

***Strain/plasmids***	***Description***	***Sources***
Strains		
*E.coli* BL21	*Res* ^−^ *Mod* ^−^ *ompT* (*DE3* with *T7 pol*) (pLysS with *T7 lysozyme*;*Cm* ^r^) Novagen	Lab stock
*E.coli* TG1	Cloning host	TaKaRa
ATCC31749	Curdlan-producing *Agrobacterium* sp. (wild-type strain)	ATCC
ATCC31749Δ*crdR*	ATCC 31749 mutant with gene knockout of *crdR*	This study
**Plasmids**		
pEGFP	GFP expression vector	Clontech
pQE81L	Expression vector, *Amp* ^R^	Qiagen
pBBR122	Gram-negative broad host vector	MoBiTec
pEX18Gm	Expression vector carrying *sac*B^R^, *Gm* ^R^	[36]
pBQ	Vector derived from both pQE80 and pBBR122	[37]
pUC19	Cloning vector	TaKaRa
pUC19T-crdR	Suicide vector for *crdR* knock-out	This study
pBQcrdR	Expression vector with *T*_*5*_ driving *crdR* expression	This study
pBQNcrdR	Expression vector with *crdRP* driving *crdR* expression	This study
pAG	Expression vector with *GFP* driven by *crd* promoter	This study
pMD18-T(*crdA)*	Derivative of pMD18-T with part of *crdA*	This study
pMD18-T*(crdS*)	Derivative of pMD18-T with part of *crdS*	This study
pMD18-T(*crdC*)	Derivative of pMD18-T with part of *crdC*	This study
pMD18-T(*crdR*)	Derivative of pMD18-T with part of *crdR*	This study

**Table 2:** **Primers used in this study**

**Primer name**	**Oligonucleotide**	**Product length**	**Product name**
*crdPG-1*	GTACTCGAGATTGTCGGCAGTCCAG	607	*crdP*
*crdPG-2*	AGCTCCTCGCCCTTGCTCACCATGAAATCAACTCCTCTGT		
GFP-1	ACAGAGGAGTTGATTTCATGGTGAGCAAGGGCGAGGAGCT	746	*GFP*
GFP-2	CGCGGATCCTTACTTGTACAGCTCGTCCATG		*crdP*
crdP-1	TCACCAACACCAACTCTGGA		
crdP-2	CATGAAATCAACTCCTCTGT	607	*crdP*
crdP142-1	ATCGTCAGATGCCTATTTGT	537	*crdP*
crdP108-1	AAATTAGTTAATGCAAT	503	*crdP*
*crdP98-1*	TTAATGCAATTTTTACTATGTT	493	*crdP*
*crdP53*-1	CCATTTCAATACTGCGGGAGG	448	*crdP*
*crdP13*-1	AGGAGTTGATTTCATGCTGTT	408	*crdP*
*crdP1*-1	ATGCTGTTCCGCAATAAG	395	*crdA*
*crdA395-2*	TCGGTCCGCAGCAGCAAAG		
*q-crdA-1*	CAAGGCATAAGCGAAGACATC	227	*crdA*
*q-crdA-2*	CTCCGTGTTTCAAGTGTGGTC		
*q-crdS-1*	AACCTGACGATTGCGATTGGG	179	*crdS*
*q-crdS-2*	GTGTAGCACCAGAGCGTTTCG		
*q-crdC-1*	GTTCGGTCAGGATGCTCAAC	248	*crdC*
*q-crdC-2*	GCCAAAGTTCGGAATCAATG		
*crdR-1*	GCCAGATCTATGACCGAGAATAAGAAAAAGCCT	437	*crdR*
*crdR-2*	TTGAGCTCTTACTCGGCGTCGCCTTCG		
*NcrdR-1*	ACACTCGAGATACACCCGGTCCCTACCAGCATT		*crdR*
*NcrdR-2*	TTTGAGCTCCTTGTCCTTCTTCAGAAGCGTGT	1302	*crdR*
*q-crdR-1*	TCGGAATGAGCCAGGAGAAGC	238	*crdR*
*q-crdR-2*	TCAGCCGAGGACAGAAAGTCG		
*crdRup-1*	TCTGAGCTCTTCGGCGTTTCGGAATGGTTG	2533	*CrdR*
*crdR*down-2	CAACACAAGCTTAACCGTCACCTGGCTCTTGGCA		
*crdR*checkGm-2	TTGGGCATACGGGAAGAAGT	558	*Gm*
*crdR*checkGm-1	CGGCTGATGTTGGGAGTAGG	577	
Rep-Kan-1	ACGCGTCGACCTTGCCAGCCCGTGGATAT	3266	*Km*
Rep-Kan-2	ACGCGTCGACTCTGTGATGGCTTCCATGTC		
Gm-1	ATAGTTGTCGAGATATTCACTAGTCGTCAGGTGGCACTTTTCG	1302	*Gm*
Gm-2	CGCGGATCCGCTCTGCTGAAGCCAGTTAC		
celAP1	ACCCGGTCTATCCCATGA		
celAP-2	CATCCAGAAACTTTCCGT	441	*celAP*
relAP-1	CCAGATTTCTCAAGGGTC		
RelAP-2	CATCATGCGATATTCCACA	443	*relAP*
crdRP-1	GCGGCGATCCTAAATGTGAC		
crdRP-2	CATGCGGTCCTGACACTCG	466	*crdRP*
crdSP-1	TCGGTCGGCACATGGGTCAAT		
crdSP-2	CATCGCCCTAACCTCGCAGT	446	*crdSP*

### Knockout of the CrdR (AGRO_0435 gene)

**Figure 1:**
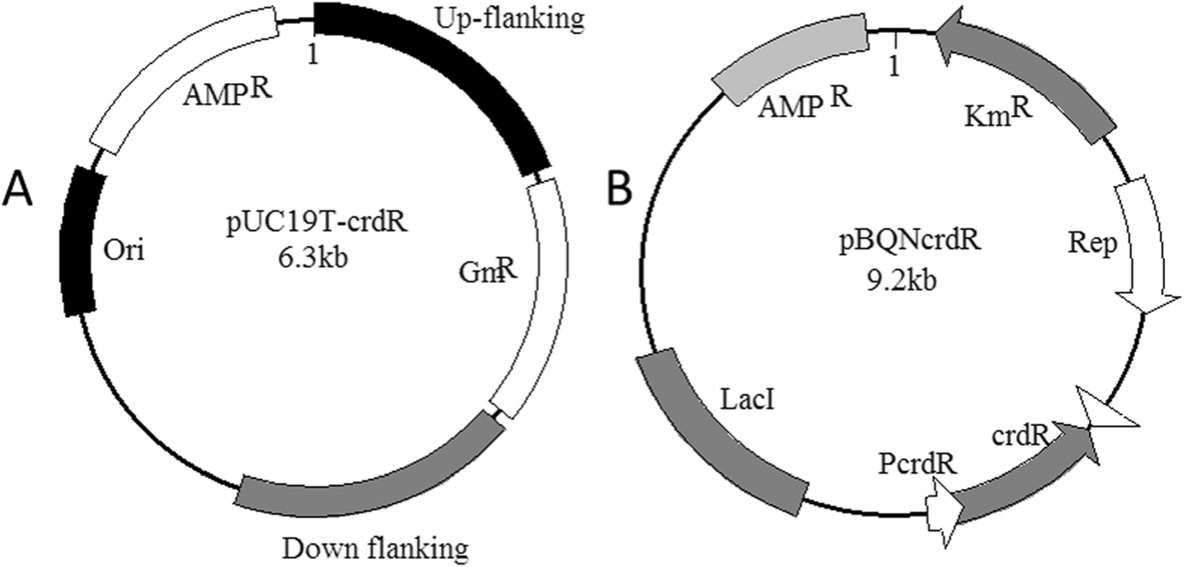
**Maps of pUC19T-crdR (A) and pBQNcrdR (B).**

For *AGRO_0435* gene knockout, a 2,533 bp fragment of the target gene (*AGRO_0435)* with up- and down-stream flanking sequences was PCR cloned using primers crdRup-1 and crdRdown-2. The amplified fragment, double digested with both *Sac*I and *Hind*III, was inserted into the same sites of pUC19 to obtain the pUCcrdR flanking. The gentamicin (Gm) resistance gene expression cassette, obtained by PCR amplification with primers (Gm-1 and Gm-2) from pEX18Gm [37], was double digested with *Bam*HI and *Sal*I and then inserted into the same sites of the pUCcrdR flanking to obtain the suicide knockout vector pUC19T-crdR (Figure 1A). Knockout plasmids were transformed with electroporation using an Eppendorf micropulser. Knockout mutants, selected by screening on LB-agar plates containing 24 μg/mL gentamicin, were confirmed with PCR amplification with 3 pairs of primers including crdR-1 and crdR-2, crdR-1 and crdRcheckGm-2, and crdRcheckGm-1 and crdR-2 (Table 2).

### Construction of pBQcrdR and pBQNcrdR for homogenous AGRO_0435 expression

A 437-bp full-length coding region of *crdR* was amplified with PCR using the primer pairs crdR-1 and crdR-2 (Table 2) with genomic ATCC31749 DNA. The amplified *crdR* fragment, digested with *BamH*I and *Sac*I, was ligated into the pBQ vector [38], creating the pBQcrdR vector (Table 1). To construct the vector for *crdR* expression driven by its native promoter *of crdR*, an *AGRO_0435* fragment with up- and down-stream flanking sequences was PCR cloned using primers NcrdR-1 and NcrdR-2 (Table 2) The obtained 1,302 bp PCR fragment which was double digested with both *Sac*I and *Xho*I was inserted into *Sal*I and *sac*I sites of pBQ to create pBQNcrdR (Table 1, Figure 1).

### Construction of pAG vector with *GFP* expression driven by the *crd* operon promoter

The predicted *crd* promoter (*crdP*), which is a 607-bp fragment upstream from the start codon of *crdA* (ATG), was amplified from genomic ATCC31749 DNA with primers crdAPG-1 and crdAPG-2 (Table 2). The *GFP* code sequence was amplified with primers GFP-1 and GFP-2 (Table 2) from plasmid pEGFP (Clontech, Mountain View, CA) and the two fragments were fused via PCR amplification. The resultant fused fragment, digested with *Xho*I and *Bam*HI, was inserted into the same sites of plasmid pQE81L to yield pQEAG. After digestion with *Sal*I, the fragment containing the gram-negative broad host replicating origin and the kanamycin (*Kan*) resistant gene amplified from pBBR122 (Table 1) with primers pairs Rep-Kan-1 and Rep-Kan-2 (Table 2), was inserted into the *Xho*I site of pQEAG to yield pAG (Table 1).

### Curdlan fermentation and yield analysis

A two-step fermentation protocol was used to measure curdlan yields. In brief, ATCC 31749 and modified strains were inoculated into test tubes containing 5 mL LB and grown overnight at 30°C with 200 revolutions per minute (rpm). About 2 mL each of the seed cultures (SC) were transferred into 500-mL flasks containing 100 mL LB with or without IPTG (final concentration 0.5 mM) at 25°C, 200 rpm for 4 h. Cells were collected by centrifugation (1000 × g for 10 min, 4°C) and cell pellets were added to 125 mL curdlan-producing medium in a 500-mL flask which was shaken at 200 rpm. Every 24 h for 5 days reaction, 15-mL samples were taken from the culture mixture and samples were centrifuged at 8,000 × g for 5 min to collect pellets. Pellets containing both cells and curdlan were resuspended in 15 mL NaOH solution (1 mol/L) for 2 h. Cells pellets were separated by centrifugation at 8,000 × g for 5 min and resulting curdlan was precipitated by the addition of 2.0 mol/L HCl and the pH was adjusted to 6.5. Curdlan was recovered by centrifugation, washed, and dried to a constant weight in an oven (80°C).

### *crdR*, *crdA*, *crdS*, and *crdC* expression analysis using q-RT-PCR

Total RNA was extracted with an EasyPure RNA Kit (TransGen Biotech, Beijing, China), according to the manufacturer’s protocol. The quality and quantity of the extracted RNA was measured using an Ultrospec 2100 spectrophotometer (Amersham Biosciences, Pittsburgh PA, USA) at 260 nm. cDNA synthesis was performed with a PrimerScript RT reagent Kit (TaKaRa, Dalian, China) according to the manufacturer’s instruction by using a 6-bp random primers set. Selected fragments of *crdR*, *crdA*, *crdS,* and *crdC*, which were amplified with primers qcrdR-1 & qcrdR-2, qcrdA-1 & qcrdA-2, qcrdS-1 & qcrdS-2, and qcrdC-1 & qcrdC-2 (Table 2), were ligated into pMD18-T vectors respectively. Then, using those constructs as standard copies, q-RT-PCR quantification was performed using an Applied Biosystems 7500 fast realtime PCR system (Applied Biosystems, Grand Island, NY ) with SYBR Premix E*TaqII (TaKaRa). All samples were run in triplicate [39].

### GFP expression

The constructed vector (pAG) was transformed into both wild-type ATCC31749 and a *crdR* mutant (ATCC31749Δ*crdR*). Transformed bacterial cells were grown in LB for 12 h and curdlan-producing medium for 72 h at 30°C. GFP expression was observed under an optical microscope (Zeiss Observer Berlin, Germany), equipped with epi-fluorescence. Simultaneously, with excitation of 450–490 nm light, Green fluorescence of GFP was measured by a fluorospectrophotometer F97Pro (FProd, Shanghai, China) to collect the data of the emission spectrum and relative fluorescence of cells harvested from both bacterial cell -growing and curdlan-producing phases.

### Expression and purification of 6 His-tagged crdR protein

6-His-tagged crdR was expressed in *E. coli* BL21 through pBQcrdR transformation. The resultant strain grew at 37°C in LB medium (OD_600_ nm = 0.5–0.6), and crdR protein expression was induced by adding IPTG (final concentration = 0.5 mM). The culture was shaken at 30°C for 4 h at 220 rpm. Cells were harvested by centrifugation were immediately extracted or frozen at −80°C until they were used. 6-His-tagged protein was purified by affinity chromatography using One-Step His-Tagged Protein Miniprep Pack (TIANDZ, Shanghai, China) according to the manufacturer’s instruction. Purified crdR dissolved in elution buffer was dialyzed with dialysis buffer (100 mM KAc, 1 mM CaCl_2,_ 1 mM MgAc_2_, 1 mM EDTA, and 1 mM dithiothreitol, 10% glycerol) overnight in a semi-permeable membrane. Protein concentration was measured using an improved Coomassie assay with bovine serum albumin (BSA) as standard.

### DNA binding analysis of CrdR by EMSA

DNA fragments containing various lengths of the *crd* promoter (*crd*P, *crd*P142, *crd*P108, *crd*P98, *crdP*53, *crd*P13, and *crdP*1) and ~450 bp upstream of the start codon (ATG) of *crdR* (relA(*AGRO_1479*) *celA* (*AGRO_4469*), and *crdS* (*AGRO_1848*) named *relAP celAP* and *crdSP* were obtained by PCR amplification with primers listed in Table 2 respectively, those fragments were purified with a DNA gel extraction kit (Sangon Biotech) respectively according to the manufacturer’s protocol. A electrophoretic mobility shift (EMSA) binding assay was performed as previously described with slight modifications [40]. Briefly, 10 μL of 0.25–0.50 mg/mL purified His-tagged crdR in 4× EMSA buffer (15 mM HEPES, 100 mM NaCl, 1 mM EDTA, 5 mM MgCl_2_, 10% glycerol, 1 μg/mL poly dI-dC) was incubated with 10 μL of different purified target DNA fragments (0.5 μM) in ddH_2_O at room temperature for 30 min. DNA-protein complexes were loaded onto a 2% agarose gel and separated at 80 V for 1.5 h, and the gel was stained with SYBR Green I and visualized with a UV trans-illuminator (Upland, CA).

## Results

### *crdR* knockout mutant construction and phenotypes

**Figure 2:**
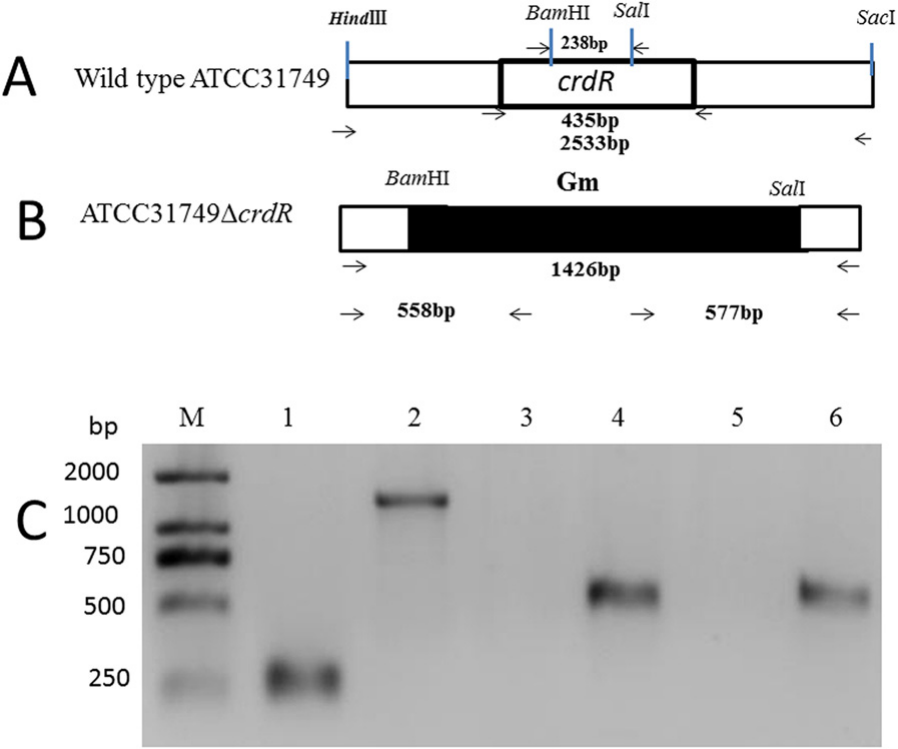
**Confirmation of the ATCC31749** ***crdR*** **mutant by PCR amplification. (A, B)** *crdR* was replaced without and with gentamicin resistance gene (*Gm*) in ATCC31749; **(C)** PCR amplification. C_M_: 2Kb DNA ladder, C_1_, C_3_, C_5_: template of genomic DNA of wild-type ATCC31749, while C_2_, C_4_, C_6_: template of genomic DNA of ATCC31749Δ*crdR* candidate were used respectively. C_2_: amplification of the *Gm* gene; C_4_: amplification of a fused fragment of the *crdR* upstream flanking region and the 5’-end of the *Gm* gene; C_6_: amplification of the fused fragment of 3’-end of the *Gm* and the *crdR* downstream flanking region.

**Figure 3:**
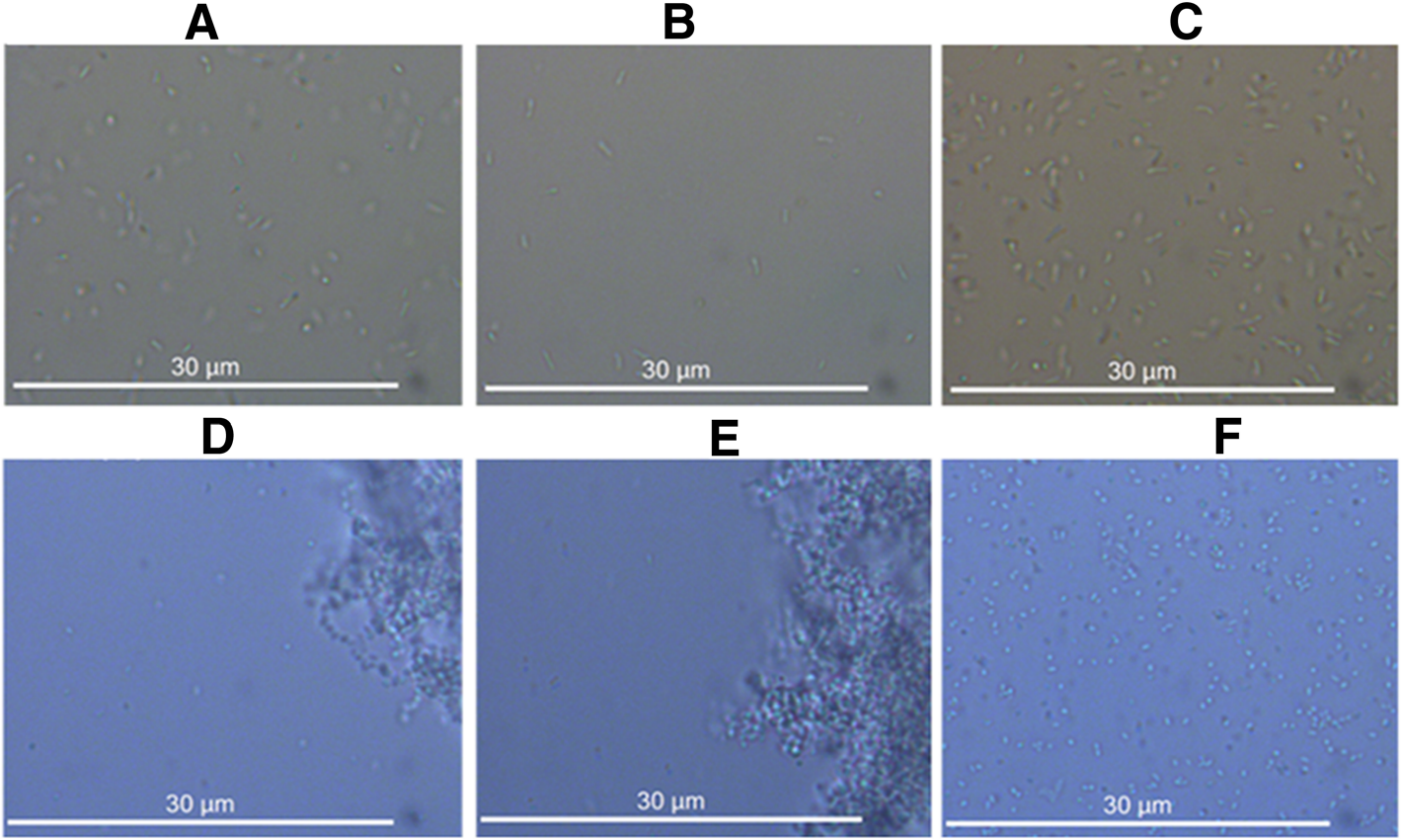
**Effects of** ***crdR*** **on morphological changes. (A, B, C)** ATCC31749, ATCC317Δ*crdR*/pBQNcrdR and ATCC317ΔcrdR cultivated in growth medium, **(D, E, F)** ATCC31749, ATCC317Δ*crdR*/pBQNcrdR, and ATCC317Δ*crdR* cultivated in curdlan-producing medium.

**Figure 4:**
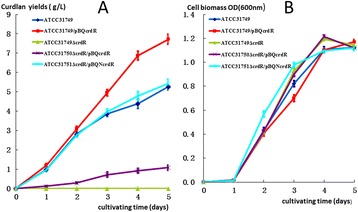
**Changes in curdlan yields (A) and cell biomasses (B) of different strains.** Data are means of 3 independent measurements; vertical bars indicate standard errors.

The *crdR* knockout mutant was constructed via homologous recombination by transformation of the suicide plasmid, pUC19T-crdR (Figure 1A). After strains were selected on gentamicin (Gm) resistant LB plates, knockout mutants were confirmed with PCR amplification (Figure 2). Compared with wild-type (WT) ATCC31749, which is capsulated in the stationary phase, the *crdR* knockout strain (ATCC31749Δ*crdR)* produced less curdlan (Figures 3 and 4) leading to motile and non-capsulated planktonic forms (Figure 3) in both exponential and stationary phases. *crdR* expression driven by promoters of *T5* and native *crdR* in both ATCC31749 and ATCC31749Δ*crd*R strains, respectively, were obtained by transforming the constructs of pBQcrdR and pBQNcrdR (Figure 1). The ATCC31749Δ*crdR*/pBQNcrdR strain recovered its curdlan capsulated form of ATCC31749 (Figure 3D and 3E). With a two-step flask-shaking process, curdlan production over 5 days [18,19] in 5 cultivated strains—ATCC31749, ATCC31749/pBQcrdR, ATCC31749Δ*crdR*, ATCC31749Δ*crdR*/pBQcrdR and ATCC31749Δ*crdR*/pBQNcrdR—was compared to assess biomass accumulation and curdlan yields. Data show that (Figure 4B) the biomass of ATCC31749Δ*crdR*, ATCC31749/pBQcrdR and ATCC31749Δ*crdR*/pBQNcrdR were higher and that ATCC31749Δ*crdR*/pBQcrdR had less accumulation than WT ATCC31749 during cultivation days 2 to 4. By the 5^th^ day, however, cell biomasses of all strains were similar. Curdlan yields for ATCC31749, ATCC31749/pBQcrdR, ATCC31749Δ*crdR*, ATCC31749Δ*crdR*/pBQ-crdR and ATCC31749Δ*crdR*/pBQNcrdR were 5.66 g/L, 7.80 g/L, 0.007 g/L, 1.13 g/L, and 5.91 g/L, respectively (Figure 4A). Curdlan yield for the *crdR* overexpressed strain (ATCC31749/pBQcrdR) was 38% greater than that of WT. *crdR* controlled by the *T5* promoter in ATCC31749Δ*crdR*/pBQcrdR could synthesize curdlan, but yields only reached 18% of WT yields; however *crdR* controlled by its native promoter in ATCC31749Δ*crdR*/pBQcrdR recovered curdlan yields of *crdR* knockout. Low curdlan yields caused by expression of *crdR* controlled with *T5* promoter suggests a more complex regulatory mechanism of *crdR* expression in the strains. Judged from these data, our observation of crdR is consistent with reports to suggest that crdR is an important regulator of curdlan biosynthesis [15,26].

### Expression of curdlan biosynthesis genes responding to *crdR* overexpression

Because *crdR* is an important regulator of curdlan biosynthesis (Figures 3 and 4), we investigated whether crdR activates expression of *crd* operon genes. q-RT-PCR analysis was used to evaluate the effects of *crdR* on *crdA*, *crdS,* and *crdC* (genes of the curdlan biosynthetic operon) mRNA. Stationary phase cells favoring curdlan biosynthesis were compared with exponential phase cells favoring cell growth, and *crdR* native expression was found to be 29.2 copies/ng total RNA in the stationery phase and 14.0 copies/ng total RNA in the exponential phase in ATCC31749/pBQ. Correspondingly, expression of *crdA*, *crdS*, and *crdC* was at least 10 times greater in the stationary phase compared to the exponential phase (Table 3). mRNA of *crdR* in ATCC31749/pBQcrdR, induced by 0.5 mM isopropyl β-D-1-thiogalactopyranoside (IPTG) at 30°C for 2 h, was significantly increased compared to that in ATCC31749/pBQ. Corresponding mRNA of *crdA*, *crdS* and *crdC* in ATCC31749/pBQcrdR were more than twice greater when strains were cultivated in both growth and fermentation media. Data confirmed that *crdR* promotes curdlan production via activating expression of *crd* operon genes (Table 3).

**Table 3:** **The expressions of the** ***crd*** genes quantified by q-RT-PCR*

	**ATCC31749/pBQ(E)**	**ATCC31749/pBQcrdR(E)**	**ATCC31749/pBQ(S)**	**ATCC31749/pBQ** ***crdR(*** **S)**
*crdA*	0.2373 ± 0.0635	1.0944 ± 0.0956	244.2038 ± 18.3645	923.5690 ± 29.6543
*crdS*	0.4050 ± 0.1022	2.7739 ± 0.1185	86.7645 ± 4.2966	176.6229 ± 9.5607
*crdC*	0.4972 ± 0.9503	2.2787 ± 0.1263	4.5239 ± 0.3371	14.5744 ± 1.0027
*crdR*	14.9112 ± 1.0359	654.8556 ± 20.4256	29.2068 ± 1.8553	193.1226 ± 11.2094

*The data are means of three independent determinations, and the unite of value is copies per ng total RNA(copies/ng RNA) E: strain in exponential phase, S:strain in stationary phase.

### *GFP* expression controlled by the *crd* operon promoter (*crdP*)

**Figure 5:**
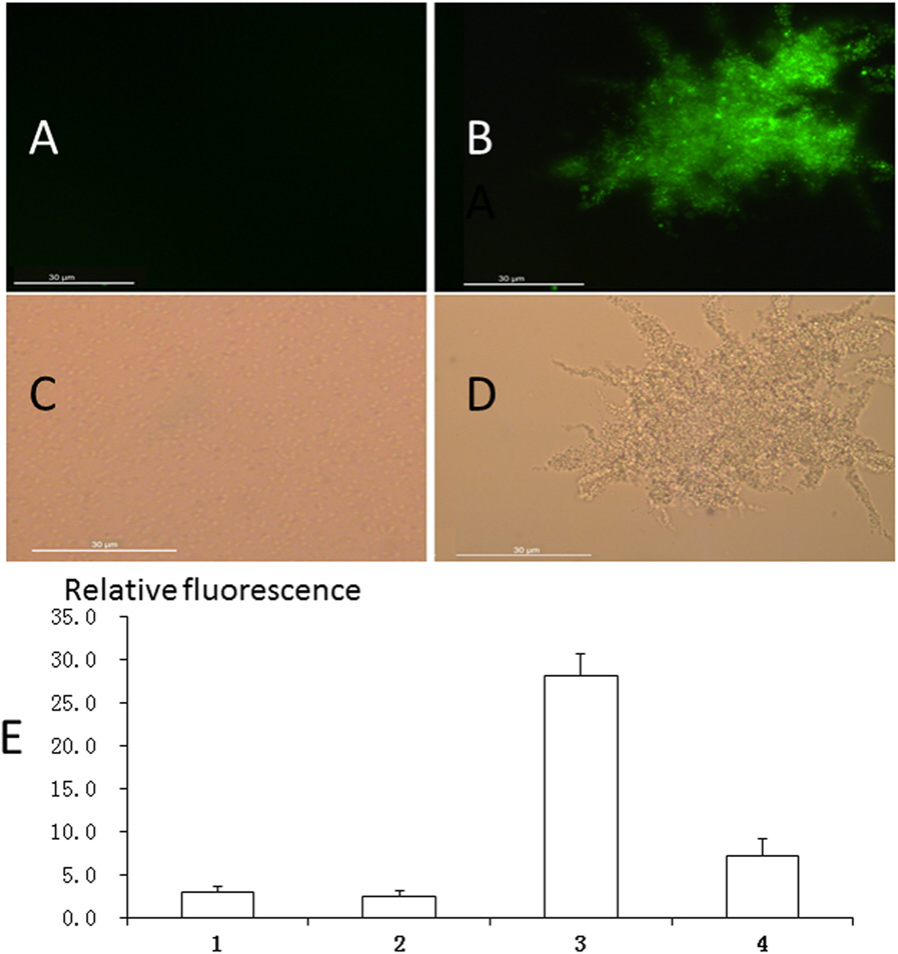
***GFP*** **expressions in constructed strains. (A, B)** Stationary phase ATCC31749Δ*crdR*/pAG and ATCC31749/pAG observed with fluorescent Microscopy; **(C, D)** Stationary phase ATCC31749Δ*crdR*/pAG and ATCC31749/pAG observed with bright-field microscopy; **(E)** relative emissive fluorescence of strains activated by light (395 nm); (E_1_, E_2_) ATCC31749/pAG and ATCC31749Δ*crdR*/pAG at exponential phase; (E_3_, E_4_) ATCC31749/pAG and ATCC31749Δ*crdR*/pAG at stationary phase. Data are means of 3 independent measurements; vertical bars indicted standard errors.

To confirm the effect of *crdR* on *crd* operon gene expression, a shuttle vector of pAG bearing *GFP* driven by the *crdP* was constructed. pAG was transformed into both ATCC31749 and its *crdR* mutant strain of ATCC31749Δ*crdR*. Data indicate that green fluorescence was undetectable by fluorescent microscopy in the *crdR* mutant strain. In contrast, strong green fluorescence was visible in the WT ATCC31749 strain grown in fermentation media (Figure 5A and 5B). Also, the ATCC31749/pAG strain was curdlan capsulated whereas ATCC31749Δ*crdR*/pAG was non-capsulated and motile (Figure 5C and 5D). *GFP* expression detected by spectrophotometry was consistent with microscopic observations that the relative GFP florescence in ATCC31749/pAG exceeded that in ATCC31749Δ*crdR*/pAG at both exponential and stationary phases (Figure 5D). Because the *GFP* expression pattern represents expression profiles of *crdA*, *crdS* and *crdC* in engineered strains of both ATCC31749/pAG and ATCC31749Δ*crdR*/pAG, the effects of *crdR* expression on *GFP* expression in those strains indicated that *crdR* might directly or indirectly interact with the *crd* operon promoter to regulate expression(s) of curdlan synthetic gene(s). Interestingly relative GFP florescence of exponential phase ATCC31749Δ*crdR*/pAG was less than that measured in the stationary phase, suggesting that *crdR* may synergistically cooperate with other regulators to control *crd* operon gene expression.

### crdR binding with’ different *crd* operon promoter regions

**Figure 6:**
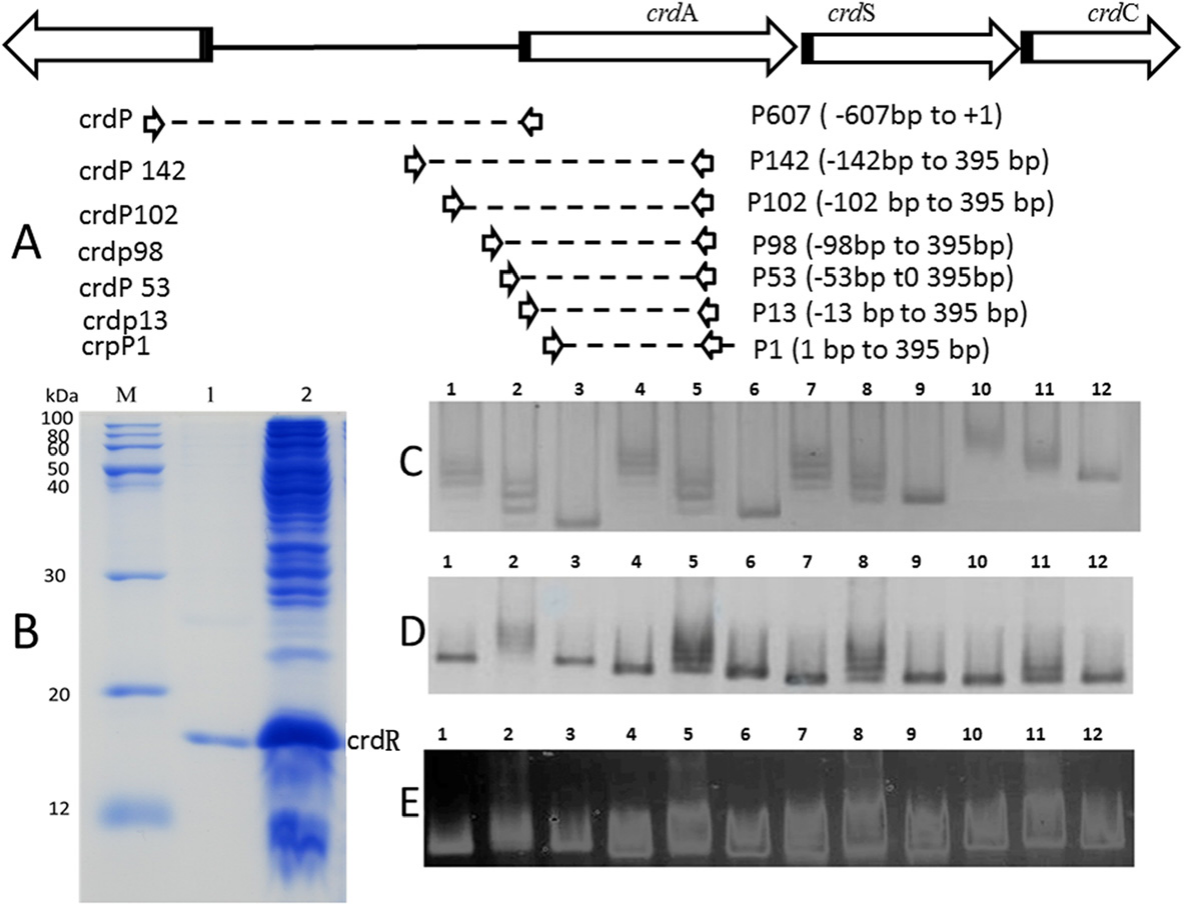
**Binding ability of 6 His-tagged crdR to different DNA fragments. A**: Different region of the *crdP*; **B**: purification of 6 His-tagged crdR; **C, D**: Binding ability 6 His-tagged crdR to different regions of *crdP;* **E**: Binding of 6 His-tagged crdR, to ~450 bp upstream of ATG at different gene coding regions. B_M_: protein markers, B_1_: purified His-tagged crdR, B_2_: supernatant of pBQcrdR/*E. coli* Bl21; C_1_. C_4_, C_7_, and C_10_ are 10 μL of 0.5 μM *crdP* 98, *crdP*102, *crdP*142 and *crdP* mixed with 10 L of 0.5 mg/mL purified 6 His-tagged crdR protein respectively, C_2_, C_5_, C_8_, and C_11_ are same as C_1_. C_4_, C_7_, and C_10_, except 6 His-tagged crdR protein was reduced to 0.25 mg/mL; C_3_, C_8_, C_9_, and C_12_ are same as C_1_. C_4_, C_7_, and C_10_, without 6 His-tagged crdR. D_1_. D_4_, D_7_, and D_10_ are 10 μL of 0.5 μM *crdP* 98, *crdP*53, *crdP*13, and *crdP*1 only respectively, D_2_, D_5_, D_8_, and D_11_ are same of .D_1_. D_4,_ D_7_, and D_10_ mixed with 10 μL 0.5 mg/mL 6 His-tagged crdR protein; D_3_, D_8_, D_9_, and D_12_ are same of .D_1_, D_4_, D_7_, and D_10_ mixed with 10 μL 0.5 mg/mL BSA respectively, E_1_, E_4_, E_7_, and E_10_ are *crdRP*, *celAP,* and *crdSP,* E_2_, E_5_, E_8_, and E_11_ are same of E_1_, E_4_, E_7_, and E_10_ mixed with 10 μL of 0.25 mg/mL 6 His-tagged crdR protein respectively, E_3_, E_6_, E_9_, and E_12_ are same of E_1_, E_4_, E_7_, and E_10_ mixed with 10 μL of 0.5 mg/mL BSA respectively.

Bioinformatic analysis of deduced amino acid sequences indicates that crdR has a conserved DNA-binding motif of a helix-turn-helix domain. To confirm that crdR protein can directly interact with the *crd* operon promoter region, with BSA as a negative protein control, DNA-binding analysis was performed with an electrophoretic mobility shift assay (EMSA) with amino terminal 6-His-tagged crdR and DNA fragments containing its putative binding sites. 6 His-tagged crdR protein was expressed and purified into a single band (15 kDa) with SDS-PAGE from *E. coli* Bl21 which was transformed with pBQcrdR (Figure 6B). The various crdR putative binding fragments, including serial regions of the *crd* promoter ranging from 607 bp upstream from ATG of *crdA* fused with or without of 395 bp downstream of ATG of *crdA* (Figure 6A) and about 450 bp upstream from ATG of *crdR*, (p) ppGpp synthetase (*relA*), cellulose synthase catalytic subunit (*celA*) and curdlan synthase catalytic subunit (*crdS*), were amplified by PCR with the genomic DNA of ATCC31749 as a template (Figure 6E). Data indicate that 6 His-tagged crdR protein cannot specifically bind 450 bp upstream of ATG of *crdR*, *relA*, *celA,* and *crdS*, but can bind to different promoter regions of the *crd* operon (Figure 6C and 6D).To locate crdR binding site(s) on the *crd* operon promoter, 607 bp upstream from ATG of the *crdA* sequence which can successfully drive *GFP* expression in the stationary phase (Figure 5), was chosen and the fragment was shorted to 98 bp upstream from the ATG of *crdA* to measure binding abilities to crdR. Data indicate that those sequences did not reduce binding to crdR as evidenced by a band shift in EMSA by mixing with or without crdR (Figure 6C). Then, the −98 bp fragments upstream from the *crdA* start codon was focused for further analysis by continual shortening to 13 bp upstream of ATG of *crdA*. Using the 395 bp coding sequence of *crdA* from 1 to 395 bp and BSA as a negative DNA and protein control, respectively, fragment mobility shifts containing −98, −53 and −13 regions of the *crd* promoter mixed with or without His-tagged crdR were observed and −98, −53 and −13 could all bind to *crdR*. However the greatest gel mobility shift was observed with the −98 fragment of *crdP*.

## Discussion

Here, we report that the *crdR*, a homolog of *PhrR* of *S. meliloti* can activate curdlan synthetic gene expression in *Agrobaterium* sp. ATCC31749. To our knowledge, ours is the first report to depict molecular functions of the *crdR* gene. Our data indicate that curdlan yield in an over-expressing *crdR* strain increased 38% compared to the WT strain. Also, pBQNcrdR transformed to the *crdR* mutant strain recovered 105% curdlan synthesis of the WT strain (Figure 4). Also, when pAG was transformed into both *crdR* mutant and WT strains *GFP* expression controlled by the *crd* promoter was undetectable by fluorescent microscopy with low relative fluorescence in the *crdR* mutant. In contrast, the WT strain had visible green color with high relative fluorescence. Finally, q-RT-PCR analysis indicated that c*rdR* is highly expressed in the stationary phase and that overexpression of *crdR* in the WT strain significantly increased expression of *crdA*, *crdS* and *crdC*. These data agree with previous reports that *crdR* is key for regulating curdlan biosynthesis. Purified crdR from *E. coli* BL21 can also specifically bind to the promoter region of *crd* offering initial evidence that crdR is a positive transcriptional regulator of the *crd* operon in ATCC31749.

The biomass accumulation in *crdR* mutant strains was not significantly different from the WT strain, suggesting that the *crdR* gene is not required for cell growth. Microscopic observation revealed that the *crdR* mutant was nearly curdlan deficient, resulting in mutant cells with non-capsulated planktonic forms. The WT ATCC31749 strain and the complementary strain of the *crdR* mutant, ATCC31749Δ*crdR*/pBQNcrdR, accumulated curdlan in the stationary phase in culture media with low pH and limited nitrogen, leading to cells were capsulated and aggregated (Figure 3). In addition, expression of *crdR* was higher in the stationary phase than in the exponential phase, and *crdR* expression further activated curdlan biosynthesis in the ATCC31749 strain to generate a biofilm. This suggests that curdlan may be critical for biofilm formation in ATCC31749 for improving stress tolerance to harsh conditions.

Bioinformatic analysis indicated that crdR can be grouped into a conserved XRE-family of transcriptional factors that is comprised of HipB in *E. coli,* PhrR in *S. meliloti*, CH00371 in *R. etli and* PraR in *A. caulinodans* (Additional file 1) [27]*.* Apparently, diverse stress can induce expressions of XRE-family transcriptional factors. Combining with HipA, HipB, a crdR homologue of *E. coli* that mediates multidrug stress tolerance can bind to its cis elements with conserved sequences of *TATCCN*_*8*_*GGATA* (where N_8_ indicates any 8 nucleotides). Genomic scanning indicated that there is no *Hip*A counterpart in the ATCC31749 strain, and that there were no conservative *TATCCN8GGATA* sequences in the promoter region of the *crd* operon. However, *crdP* does have three distinct hairpin structures located at the −10, −35, and −92 regions (Additional file 2), which are putative crdR binding sites. That purified crdR can bind to an amplified fragment containing the −92 region of c*rdP* more than the −53, and −13 regions indicates that those region are likely the crdR binding site. HipAB is a heterodimer of a transcriptional repressive regulator [28], and crdR may play a different role as a transcriptional activator in the form of a homotetramer or homodimer, which must be confirmed with additional studies. *PhrR* in *S. meliloti* was affected by low pH, Cu^2 +^, Zn^2 +^ and H_2_O_2_ stresses [26]. Expression of *CH00371* in *R. etli* was promoted by oxidation and osmotic shock [29,30]; whereas expression of *PraR* in *A. caulinodans* was increased by low nitrogen [31]. Primary data from transcriptome analysis obtained from RNAseq indicates that expression of *crdR* in the curdlan fermenting phase with both low pH and limited nitrogen was twice as great as that in the growing phase of bacterium in LB medium (data not shown) and q-RT-PCR analysis of *crdR* expression agreed with RNA-Seq data (Table 3). Thus, *crdR* expression should be triggered by stress factors as well.

Most organisms within the genera of *Rhizobium*, *Azorhizobium*, *Bradyrhizobium*, *Mesorhizobium*, and *Sinorhizobium* are symbiotic bacteria to various leguminous plants [32,33]. After bacteria enter plant tissues, their environment changes to low pH (normally 5.5), with limited nitrogen and sufficient carbohydrates [32,33], and this environmental shift affects strain morphology and physiology. To survive with limited nitrogen, genes related to bacterial nitrogen fixing and those for low pH tolerance are expressed. Transcriptional factors such as PhrR and its homologues are instrumental for expression of low pH tolerance genes. Also, with abundant carbohydrates from host photosynthesis, bacterial extracellular polysaccharides (EPS) are synthesized to produce a biofilm [34] to protect the bacteria from stress. Also, the biofilm provides a low-oxygen environment inside the bacteria to support nitrogen fixing reactions. This concept is in agreement with research with *PraR* from *A. caulinodans* ORS571, a homolog of *crdR*, which can mediate stem nodule formation, regulate expression of *Reb* genes, and increase nitrogen fixation of bacterial strains within stem nodules [31]. Therefore, biosynthesis of curdlan regulated by *crdR* may originate from ancestral characteristics for survival within a host plant. Currently, detailed regulatory mechanisms of *crdR* expression, controlled by disadvantageous conditions such as low pH and limited nitrogen remain unknown. However, reports regarding stringent response signal(p)ppGpp, which can induce EPS biosynthesis and bacterial biofilm formation [35] is worthy of study. We hypothesize that *crdR* expression may be activated by (p)ppGpp, which accumulates under stressful conditions.

## Conclusions

In this study, we confirmed that crdR regulates curdlan synthesis by activating expressions of its biosynthetic genes. Ours is the first work to identify XER family transcriptional factor which can activate EPS biosynthesis. crdR may be a multiple-effect regulator controlling expression(s) of the curdlan synthesis gene(s) in ATCC31749 under oxidative stress/low pH and/or limited nitrogen with abundant sugar. This function of curdlan regulation indicates that curdlan biosynthesis of ATCC31749 under harsh conditions may have evolutionary origin.

## Additional files

Additional file 1:
**Comparative analysis of crdR homologous protein.** Protein sequences of crdR homologous were compared clustalX2 software and PhrR (with 90% similarity to crdR, the same as below) from *S. meliloti*, CH00371 (86%) from *R. leguminosarum*, PraR (57%) from *A. Caulinodans*. Additional file 2:
**A98 pb promoter sequence of the** ***crd*** **operon (** ***crdP*** **) upstream from starting codon of** ***crdA*** **(ATG) with its secondary structure predicted by DNAMAN 98 bp fragment upstream ATG) of the** ***crdA*** **have 3 putative crdR binding sites, which has 3 hairpin structures located in −92, −35, and −10 regions.**
