# Supplementary figures and images for: CrdR function in a curdlan-producing Agrobacterium sp. ATCC31749 strain
Source: BMC Microbiol. 2015 Feb 10;15(1):25. doi: 10.1186/s12866-015-0356-1 (PMC4327974; doi:10.1186/s12866-015-0356-1)

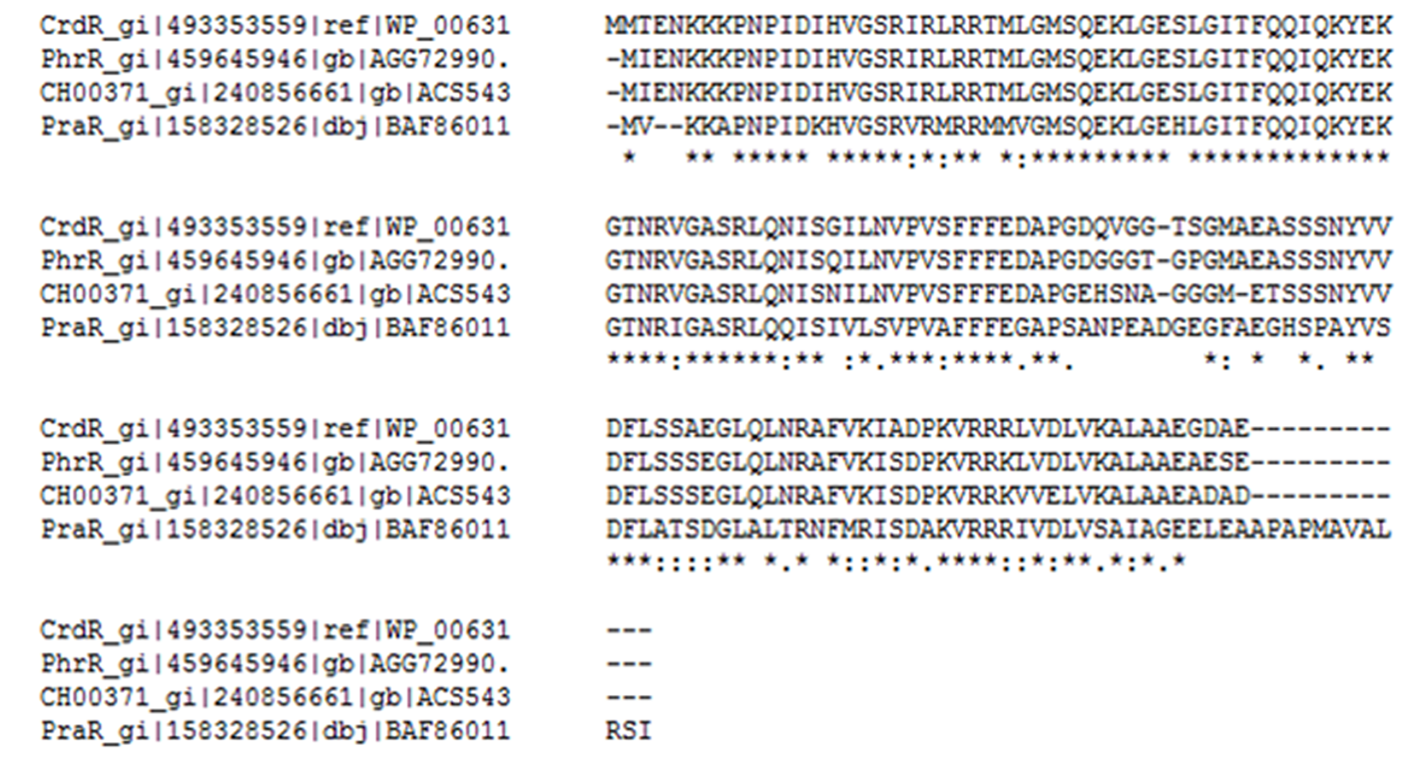

Supplement: Additional file 1: — Comparative analysis of crdR homologous protein. Protein sequences of crdR homologous were compared clustalX2 software and PhrR (with 90% similarity to crdR, the same as below) from S. meliloti, CH00371 (86%) from R. leguminosarum, PraR (57%) from A. Caulinodans. [file 12866_2015_356_MOESM1_ESM.tiff]

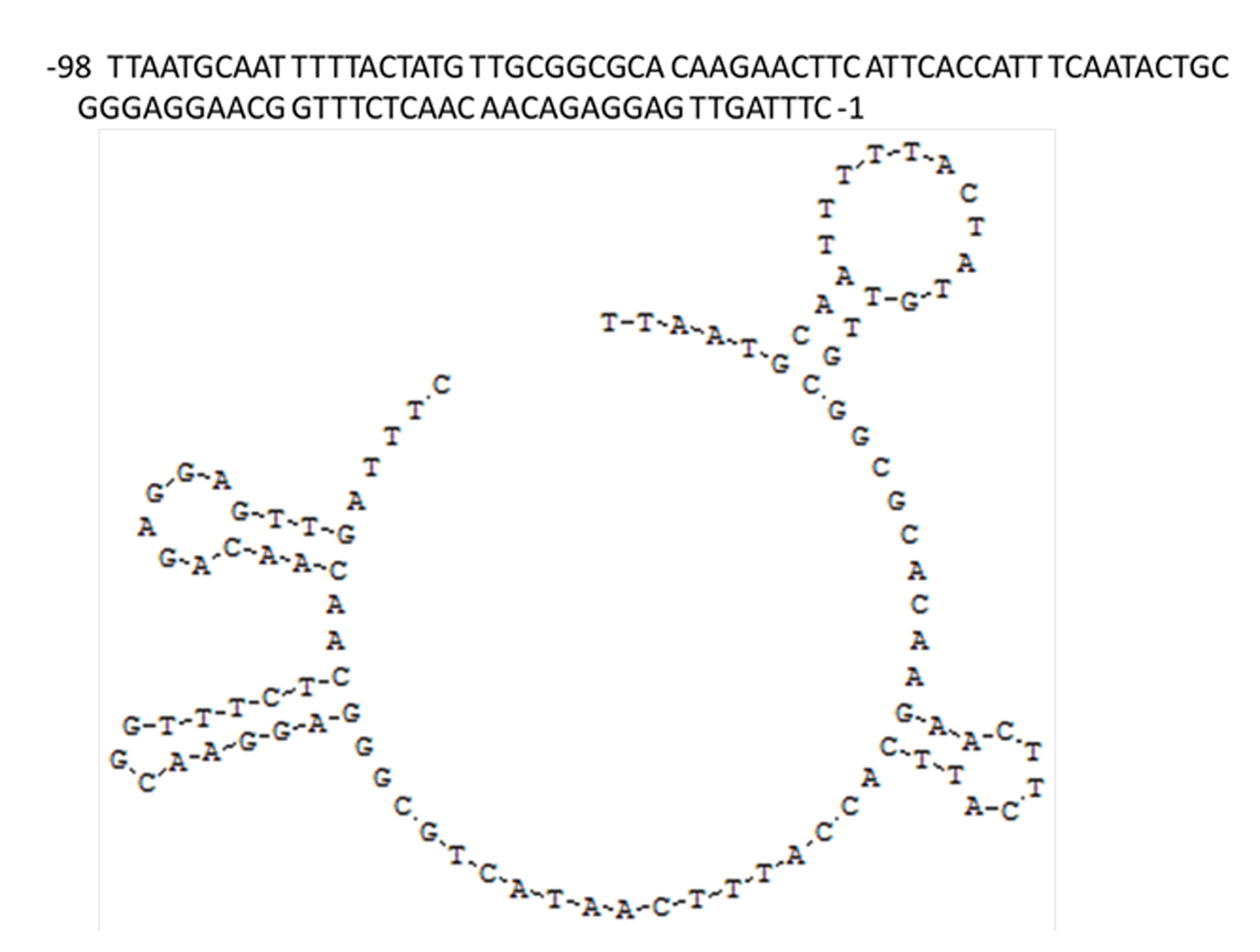

Supplement: Additional file 2: — A98 pb promoter sequence of the crd operon ( crdP ) upstream from starting codon of crdA (ATG) with its secondary structure predicted by DNAMAN 98 bp fragment upstream ATG) of the crdA have 3 putative crdR binding sites, which has 3 hairpin structures located in −92, −35, and −10 regions. [file 12866_2015_356_MOESM2_ESM.tiff]
